# Effects of lung protective mechanical ventilation associated with permissive respiratory acidosis on regional extra-pulmonary blood flow in experimental ARDS

**DOI:** 10.1186/s12871-017-0439-7

**Published:** 2017-10-27

**Authors:** Rudolf Hering, Stefan Kreyer, Christian Putensen

**Affiliations:** 1 Department of Anesthesia and Intensive Care Medicine, University Hospital of Bonn, Bonn, Germany; 2 Department of Anesthesia, Intensive Care, Emergency and Pain Medicine, Kreiskrankenhaus Mechernich GmbH, Mechernich, Germany

**Keywords:** Cardiac output, Lung protective ventilation, Respiratory distress syndrome, Regional blood flow, Hemodynamics, Permissive hypercapnia

## Abstract

**Background:**

Lung protective mechanical ventilation with limited peak inspiratory pressure has been shown to affect cardiac output in patients with ARDS. However, little is known about the impact of lung protective mechanical ventilation on regional perfusion, especially when associated with moderate permissive respiratory acidosis. We hypothesized that lung protective mechanical ventilation with limited peak inspiratory pressure and moderate respiratory acidosis results in an increased cardiac output but unequal distribution of blood flow to the different organs of pigs with oleic-acid induced ARDS.

**Methods:**

Twelve pigs were enrolled, 3 died during instrumentation and induction of lung injury. Thus, 9 animals received pressure controlled mechanical ventilation with a PEEP of 5 cmH_2_O and limited peak inspiratory pressure (17 ± 4 cmH_2_O) versus increased peak inspiratory pressure (23 ± 6 cmH_2_O) in a crossover-randomized design and were analyzed. The sequence of limited versus increased peak inspiratory pressure was randomized using sealed envelopes. Systemic and regional hemodynamics were determined by double indicator dilution technique and colored microspheres, respectively. The paired student t–test and the Wilcoxon test were used to compare normally and not normally distributed data, respectively.

**Results:**

Mechanical ventilation with limited inspiratory pressure resulted in moderate hypercapnia and respiratory acidosis (PaCO_2_ 71 ± 12 vs. 46 ± 9 mmHg, and pH 7.27 ± 0.05 vs. 7.38 ± 0.04, *p* < 0.001, respectively), increased cardiac output (140 ± 32 vs. 110 ± 22 ml/min/kg, p<0.05) and regional blood flow in the myocardium, brain and spinal cord, adrenal and thyroid glands, the mucosal layers of the esophagus and jejunum, the muscularis layers of the esophagus and duodenum, and the gall and urinary bladders. Perfusion of kidneys, pancreas, spleen, hepatic arterial bed, and the mucosal and muscularis blood flow to the other evaluated intestinal regions remained unchanged.

**Conclusions:**

In this porcine model of ARDS mechanical ventilation with limited peak inspiratory pressure resulting in moderate respiratory acidosis was associated with an increase in cardiac output. However, the better systemic blood flow was not uniformly directed to the different organs. This observation may be of clinical interest in patients, e.g. with cardiac, renal and cerebral pathologies.

**Electronic supplementary material:**

The online version of this article (10.1186/s12871-017-0439-7) contains supplementary material, which is available to authorized users.

## Background

Lung protective mechanical ventilation has been shown to improve the outcome of patients with ARDS [1]. Beyond the beneficial effects of limiting peak inspiratory airway pressures (P_AW_) and tidal volumes (V_T_) on the lungs by reducing stress and strain [2], alveolar cyclic recruitment-derecruitment [3], and pulmonary and systemic inflammatory responses [4] the impact of mechanical ventilation on the cardiovascular system is of growing interest. A group of experts recently stated that successfully managing the complex hemodynamics of the ventilated patient with ARDS is a key to patient survival [5]. Actually, ARDS and the strategy of mechanical ventilation not only affects the right heart and pulmonary vascular system [6] but also systemic blood flow [7–9]. Natalini et al. recently demonstrated that lung protective ventilation and moderate hypercapnia resulted in an improvement in cardiac output (CO) and a decrease in oxygen extraction ratio in ARDS patients [10]. Extending these results of improved systemic hemodynamics to the regional vascular beds would be of great interest.

Because measuring organ and tissue perfusion directly is not feasible in critically ill patients, we conducted an experimental study using pigs with oleic-acid induced ARDS which were mechanically ventilated in a pressure controlled mode either with limited inspiratory P_AW_ targeting moderate respiratory acidosis or increased inspiratory P_AW_ targeting normal pH. Regional perfusion of the myocardium, brain and spinal cord, kidneys, liver, spleen, pancreas, intestinal mucosa and muscularis, gall and urinary bladder, adrenal and thyroid glands were determined with colored microspheres. We hypothesized that limiting inspiratory P_AW_ associated with moderate respiratory acidosis results in an increase in systemic blood flow but an unequal distribution of blood flows to different organs and tissues.

## Methods

### Animal care approval

The experiments were performed in accordance with German legislation governing animal studies and the International Association of Veterinary Editors’ Consensus Author Guidelines on Animal Ethics and Welfare [11]. The study was reviewed by a governmental ethical committee and official permission was granted from the governmental animal care and use committee of the Regional Government of the District of Cologne, Germany (23.203.2-BN43, 15/00).

### Sample size

We knew from previous studies of our group that the number of experimental animals was 12 including a drop-off rate of approximately 20 to 30% to detect differences in systemic and regional blood flows between different ventilatory settings with a given two-sided crossover design at a significance level of 5% (α < 0.05) with a probability of 80% (β = 0.20) [12, 13]. Therefore, we a priori determined to use twelve pigs, mixed German breed, weighing 12 to 20 kg (16.3 ± 1.83 kg; mean ± standard deviation (SD)).

### Instrumentation

Before instrumentation, the animals were premedicated with intramuscular ketamine (10 mg/kg), xylazine hydrochloride (2 mg/kg), and placed supine on a heating pad to maintain core temperature at 38 °C. Anesthesia was induced with intravenous sodium pentobarbital, a bolus of 10 mg/kg, followed by infusion of 2 mg/kg/h and 2 mg/kg/h ketamine. The dosage of anesthetics was not changed until the end of the study, when the animals were killed using an overdose of pentobarbital and potassium chloride. To ensure adequate hydration 500 ml Ringer solution was rapidly infused followed by an infusion rate of 5 ml/kg/h throughout the study. Animals were intubated with a 7.5 mm internal diameter cuffed tracheal tube and ventilated using pressure controlled mechanical ventilation (Evita 2, Dräger Inc. Lübeck, Germany).

A 4-French thermistor-tipped pulmonary artery catheter (AI-07044; Arrow International, Inc., Reading, PA) was inserted through the right jugular vein. The left carotid artery was cannulated and a 4-Fr pigtail catheter (Duct-Occlud 147,420; pfm, Germany) was inserted into the left ventricle for injection of microspheres. A 7-French 3-lm catheter (OW-14703-E; Arrow International, Inc., Reading, PA) was placed in the abdominal aorta via the right femoral artery for pressure monitoring and blood sampling, and a 4-French thermistor-tipped fiberoptic catheter (Pulsiocath PV2024L, Pulsion Medical Systems, Munich, Germany) was advanced through the left femoral artery into the descending thoracic aorta. At the end of each experiment, the correct position of all catheters was verified by autopsy.

### Ventilatory measurements

Gas flow was measured at the proximal end of the tracheal tube with a heated pneumotachograph (No.2, Fleisch, Lausanne, Switzerland) connected to a differential pressure transducer (Huba Control, Würenlos, Switzerland). Respiratory rate, V_T_, and minute ventilation were derived from the integrated gas flow signal. Airway pressure was measured at the proximal end of the tracheal tube with a differential gas-pressure transducer (SMT, Munich, Germany). Data were sampled via an analog-digital converter (DT 2801-a; Data-Translation, Marlboro, MA) at sampling intervals of 3 s, processed, and stored on a personal computer for offline-analysis. The software for data acquisition and evaluation was programmed using a commercially available software tool (Asyst®4.0; Keithly Asyst; Taunton, MA).

### Cardiovascular measurements

Heart rate was obtained from the electrocardiogram. Systemic mean arterial pressure, central venous, mean pulmonary artery, and pulmonary artery occlusion pressures were transduced (Combitrans, Braun AG, Melsungen, Germany), recorded (CS/3, Datex, Helsinki, Finland), and stored on a personal computer. The transpulmonary double-indicator dilution method was used to estimate CO and intrathoracic blood volume as described previously [14]. Indocyanine green dye (Becton Dickinson, Cockeysville, MD, USA), 3 mg dissolved in 6 ml iced 5% glucose solution was used as double-indicator and injected into the superior vena cava. Simultaneously dilution curves for dye and temperature were recorded in the aorta with the thermistor-tipped fiberoptic artery catheter. From these curves a computer (COLD-Z-021, Pulsion Medical Systems, Munich, Germany) estimated CO with the dye-dilution method and determined the mean transit time of the first pass of the dye indicator for calculating intrathoracic blood volume [14, 15]. Three determinations were performed at random moments during the ventilatory cycle and averaged. Standard formula were used to calculate systemic and pulmonary vascular resistance, and oxygen delivery. Values were indexed for body weight if appropriate.

### Blood gas analysis

Arterial PCO_2_ and pH were determined with standard blood gas electrodes (ABL 510, Radiometer, Copenhagen, Denmark). Oxygen saturation (SO_2_) of arterial and mixed venous blood, and total hemoglobin were determined by spectrophotmetry with a CO-oximeter (OSM3, Radiometer, Copenhagen, Denmark).

### Tissue blood flow measurements

The colored microsphere technique was used to measure regional blood flow as described previously [12]. The polystyrene microspheres (Dye Track; Triton Technology, San Diego, CA) are coated with a single colored dye and are 15 ± 0.3 μm SD in diameter. Red-, blue-, yellow-, white- or violet microspheres were used. Depending on the different absorbance characteristics of each color, 6 to 15 million microspheres suspended in 2 to 5 ml of 0. 9% saline solution containing 0.02% Tween 80 were used for each blood flow measurement. Starting 10 s before injection of the microspheres into the left ventricle and continuing for 120 s after injection was completed, two reference blood samples were withdrawn simultaneously from different lumina of the aortic catheter at a rate of 5 ml/min with a precision pump (AH 55–2226; Harvard Apparatus GmbH; March-Hugstetten, Germany). At the end of the experiments, animals were killed with sodium pentobarbital and potassium chloride. All organs and tissues to be evaluated were removed and cut into small pieces weighing 1–2.5 mg.

The trapped microspheres in each tissue- and blood samples were quantified by their dye content. After digestion of the tissue/blood with 4 M potassium hydroxide microspheres were harvested on a polyester filter (pore size: 8 μm, diameter: 25 mm, Nucleopore; Costar; Bodenheim; Germany) and washed with 2% Tween 80 and then with ethanol. The dye-cover was solved from the microspheres by using 200 μl of dimethylformamide. Then, the dye-suspension was separated from the microspheres by centrifugation. Spectrophotometric analysis of mixed dye solutions was performed (Spectrophotometer DU64; Beckmann; Düsseldorf; Germany) and the composite spectrum of each dye solution was resolved into spectra of single constituents using a matrix inversion software package (Dye-Track; Triton Technologies; San Diego; CA). From spectrophotometric data tissue blood flow was calculated by the formula: Tissue blood flow (ml· g^−1.^ min^−1^) = As· Vref· Aref^−1.^ Ws^−1^ where As is absorbance of the tissue sample, Vref is the reference blood flow (ml· min^−1^), Aref is the mean absorbance of both reference blood samples, and Ws is the weight of the tissue sample. For each organ, the respective median blood flow of all samples was calculated.

### Experimental protocol

During instrumentation the animals were ventilated with a positive end-expiratory pressure set at 5 cm H_2_O, a peak inspiratory P_AW_ set at 15 cmH_2_O, an inspiratory to expiratory ratio set at 1:1, and an inspiratory oxygen fraction (FiO_2_) of 30%. Experimental ARDS was induced by injection of 0.1 ml/kg purified oleic acid (J.T. Baker Inc., Phillipsburg, NJ) into the right atrial catheter over 30 min. Additional 0.2 ml increments of oleic acid were administered every 30 min until PaO_2_ was less than 50 mmHg. FiO_2_ was increased to avoid SaO_2_ below 85% and remained unchanged thereafter.

After induction of ARDS pigs were paralyzed using atracurium and mechanically ventilated using either an unchanged peak inspiratory P_AW_ limited to 15 cm H_2_O resulting in moderate respiratory acidosis or an increased peak inspiratory P_AW_ to compensate for respiratory acidosis. The sequence of ventilation with limited or increased inspiratory P_AW_ was randomized using sealed envelopes. During ventilation with limited inspiratory P_AW_, respiratory rate and FiO_2_ were allowed to be adjusted to maintain SaO_2_ ≥ 85% and pH above 7.25. If the respiratory rate had to be increased to an extent that inspiratory or expiratory flow curves did not return to zero at the end of inspiration or expiration indicating too short in- and deflation times the respiratory rate was reduced and P_AW_ adjusted to increase V_T_ in steps of 1 ml/kg to assure sufficient oxygenation (SaO_2_ ≥ 85%) and decarboxylation (pH ≥ 7.25). Thirty minutes of equilibration were allowed for each intervention before measurements.

### Statistical analysis

Values are presented either as mean ± SD or as median (lower quartile; upper quartile). Data were evaluated for normal distribution with the Shapiro-Wilks W test. If values were normal distributed data obtained during the different ventilatory strategies were compared using the paired student t–test, if data were not normally distributed data were compared using Wilcoxon test. To verify adequate mixing of microspheres in the blood circulation and even distribution of blood flow to the various organs after injection into the left ventricle, correlations were calculated between the numbers of microspheres trapped in the two reference blood samples as well as between blood flows to the right and left adrenal glands by using general linear regression. Differences were considered to be statistically significant if p was less than 0.05.

## Results

Of the 12 pigs included in the study one animal died during instrumentation due to refractory ventricular fibrillation after introducing the left ventricular pig-tail catheter, and two pigs died due to hypoxemia and right ventricular failure during induction of ARDS. Thus, data of 9 pigs were included into the analysis. Of these animals, 5 started with increased peak inspiratory P_AW_ and 4 with limited inspiratory P_AW_. Before measurements each of the ventilation settings reached its steady state.

Adequate mixing and distribution of injected microspheres was verified by highly significant correlations between the trapped number of microspheres in the two reference blood samples and the blood flow to the right and left adrenal gland (*r* = 0.98, *p* < 0.0001).

Peak inspiratory P_AW_ was increased to 23 ± 6 cm H_2_O during ventilation with increased P_AW_ as compared to 17 ± 4 cm H_2_O during ventilation with limited P_AW_. This resulted in significantly increased mean P_AW_, V_T_, minute ventilation, and pH and a decrease in PaCO_2_ during ventilation with higher inspiratory P_AW_ (*p* < 0.001, respectively). The increase in respiratory rate and FiO_2_ during ventilation with limited P_AW_ to maintain pH above 7.25 and SaO_2_ above 85% were not statistically significant. During ventilation with limited P_AW_ PaO_2_/ FiO_2_ (*p* < 0.05) decreased (Table 1).

**Table 1:** Ventilation and gas exchange variables

	Increased P_AW_	Limited P_AW_	
FiO_2_, %	66 ± 25	84 ± 21	n.s.
Positive end-expiratory pressure, cm H_2_O	5 ± 0	5 ± 0	n.s.
Peak inspiratory pressure, cm H_2_O	23 ± 6	17 ± 4	*p* < 0.001
Mean airway pressure, cm H_2_O	14.8 ± 2.6	11.4 ± 1.8	*p* < 0.001
V_T_, mL. kg^−1^	9.5 ± 3.0	6.2 ± 2.9	*p* < 0.001
Minute ventilation, L. min^−1^	4.9 ± 0.8	3.4 ± 1.2	*p* < 0.001
Respiratory rate, min^−1^	31 ± 2	34 ± 3	n.s.
PaO_2_ / FiO_2_, mm Hg	231 ± 104	182 ± 90	*p* < 0.05
PaCO_2_, mm Hg	46 ± 12	71 ± 9	*p* < 0.001
pH_a_	7.38 ± 0.04	7.27 ± 0.05	*p* < 0.001

*P*_*AW*_ airway pressure, *FiO*_*2*_ inspiratory fraction of oxygen, *V*_*T*_ tidal volume. Values are presented as mean ± standard deviation (SD); tested on a randomized basis; student t-test

Limiting P_AW_ was associated with an increase in CO, mean arterial and pulmonary arterial pressure, and SvO_2_ (*p* < 0.05, respectively) while heart rate, central venous and pulmonary capillary wedge pressure, intrathoracic blood volume, systemic and pulmonary vascular resistance, stroke volume, SaO_2_, oxygen delivery, and lactate remained unchanged (Table 2).

**Table 2:** Cardiovascular variables

	Increased P_AW_	Limited P_AW_	
Heart rate, min^−1^	115 ± 35	137 ± 46	n.s.
Mean arterial pressure, mm Hg	98 ± 16	117 ± 16	*p* < 0.05
Mean pulmonary artery pressure, mmHg	28 ± 9	32 ± 11	*p* < 0.05
Pulmonary capillary wedge pressure, mm Hg	8 ± 3	7 ± 3	n.s.
Central venous pressure, mm Hg	12 ± 5	11 ± 4	n.s.
Intrathoracic blood volume, mL ^.^ kg^−1^	20.7 ± 4.2	23.1 ± 4.2	n.s.
Cardiac output, mL. kg^−1^. min^−1^	110 ± 22	140 ± 32	*p* < 0.05
Stroke volume, mL. kg^−1^. beat^−1^	1.04 ± 0.35	1.08 ± 0.25	n.s.
Systemic vascular resistance, mm Hg. kg. min. mL^−1^	2339 ± 571	2329 ± 625	n.s.
Pulmonary vascular resistance, mm Hg. kg. min. mL^−1^	555 ± 237	532 ± 218	n.s.
Hemoglobin, g. dL^−1^	9.9 ± 1.5	10.7 ± 1.5	*p* < 0.05
SaO_2_, (%)	94 ± 9	90 ± 11	n.s.
SvO_2_ (%)	50 ± 12	62 ± 13	*p* < 0.05
Oxygen delivery, mL. kg^−1^. min^−1^	13.0 ± 2.7	13.4 ± 2.4	n.s.
Lactate (mmol/l)	1 ± 0.4	0.8 ± 0.4	n.s.

*P*_*AW*_ airway pressure, *SaO*_*2*_ arterial oxygen saturation, *SvO*_*2*_ mixed venous oxygen saturation. Values are presented as mean ± standard deviation (SD); tested on a randomized basis; student t-test

**Fig. 1:**
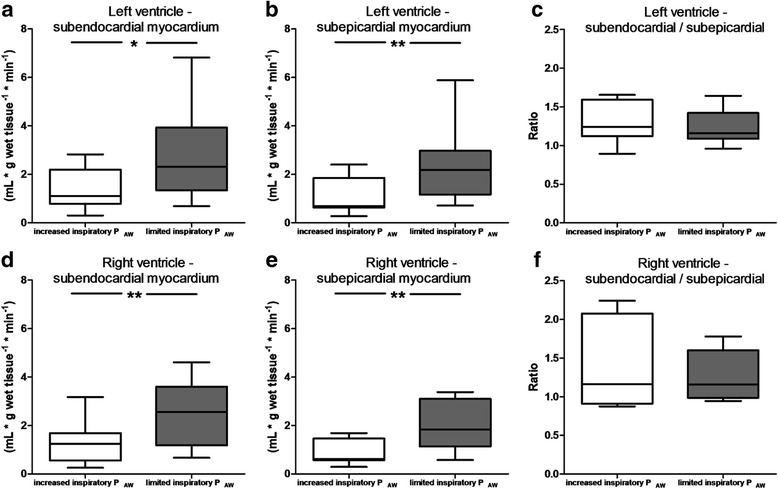
Myocardial perfusion during mechanical ventilation with limited peak inspiratory pressure targeting permissive respiratory acidosis and increased peak inspiratory pressure targeting normal pH. Panel **a**: left ventricle - subendocardial myocardium; panel **b**: left ventricle - subepicardial myocardium; panel **c**: left ventricle - ratio of subendocardial to subendocardial perfusion; panel **d**: right ventricle - subendocardial myocardium; panel **e**: right ventricle - subepicardial myocardium; panel **f**: right ventricle - ratio of subendocardial to subendocardial perfusion; Boxes are median and 25/75 percentile, whiskers are 5/95% percentile. * *p* < 0.05 compared with increased P_AW_; ** *p* < 0.01 compared with increased P_AW_

**Fig. 2:**
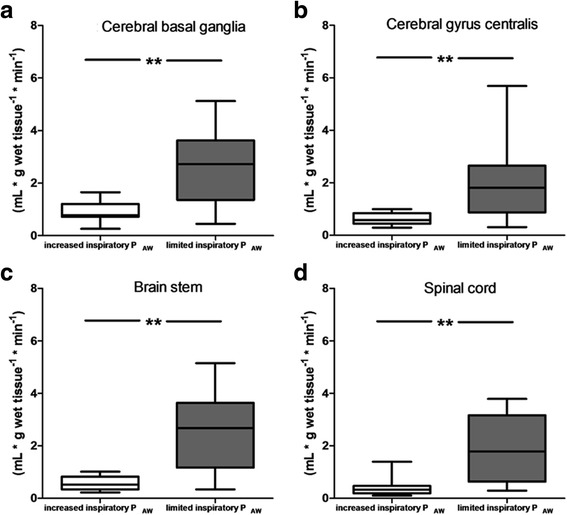
Regional cerebral and spinal cord perfusion during mechanical ventilation with limited peak inspiratory pressure targeting permissive respiratory acidosis and increased peak inspiratory pressure targeting normal pH. Panel **a**: cerebral basal ganglia; panel **b**: cerebral gyrus centralis; panel **c**: brain stem; panel **d**: spinal cord. Boxes are median and 25/75 percentile, whiskers are 5/95% percentile. ** *p* < 0.01 compared with increased P_AW_

During ventilation with limited inspiratory P_AW_ and moderate respiratory acidosis the increase in CO resulted in a significant increase in blood flow both to the subendocardial and subepicardial myocardium of the right and left ventricle while the ratio of subendocardial to subepicardial perfusion remained unchanged in both ventricles (Fig. 1). Regional blood flow to the brain and spinal cord also increased during ventilation with limited inspiratory P_AW_ (Fig. 2). Similarly, blood flow increased to the adrenal (*p* < 0.01) and thyroid glands (*p* < 0.05) (Table 3), the mucosal layers of the esophagus (*p* < 0.01) and jejunum (*p* < 0.05), the muscularis layers of the esophagus and duodenum (*p* < 0.05, respectively), and the gall and urinary bladders (*p* < 0.01, respectively) (Table 4). Perfusion of kidneys, pancreas, spleen, hepatic arterial bed, and the mucosa and muscularis layers of the other evaluated intestinal regions remained unchanged (Tables 3, 4).

**Table 3:** Regional blood flow to solid organs (ml ^.^ g wet tissue^−1 .^ min^−1^)

	Increased P_AW_	Limited P_AW_	
Kidneys	1.85 (1.36; 2.12)	2.02 (1.53;3.78)	n.s.
Adrenal glands	1 (0.76; 1.64)	2.94 (2.14;7.89)	*p* < 0.01
Thyroidal gland	0.16 (0.09;0.26)	0.25 (0.17;0.68)	*p* < 0.05
Spleen	1.58 (1.31;3.16)	2.91 (1.38;6.32)	n.s.
Pancreas	0.18 (0.16;0.32)	0.23 (0.20;0.31)	n.s.
Liver (arterial vascular bed)	0.5 (0.22;0.72)	0.47 (0.31;0.95)	n.s.

*P*_*AW*_ airway pressure. Values are presented as median (lower quartile; upper quartile); tested on a randomized basis, Wilcoxon matched pairs test

**Table 4:** Intestinal and urinary bladder blood flow (ml ^.^ g wet tissue^−1 .^ min^−1^)

	Increased P_AW_	Limited P_AW_	
Esophagus (mucosa-submucosa)	0.18 (0.10; 0.31)	0.32 (0.26; 0.41)	*p* < 0.01
Esophagus (muscularis-serosa)	0.09 (0.06; 0.14)	0.16 (0.15; 0.21)	*p* < 0.05
Stomach (mucosa-submucosa)	0.16 (0.14; 0.27)	0.21 (0.16;0.29)	n.s.
Stomach (muscularis-serosa)	0.05 (0.03; 0.08)	0.09 (0.05; 0.11)	n.s.
Duodenum (mucosa-submucosa)	0.44 (0.39; 0.52)	0.58 (0.3; 0.73)	n.s.
Duodenum (muscularis-serosa)	0.1 (0.05; 0.17)	0.16 (0.08;0.3)	*p* < 0.05
Jejunum (mucosa-submucosa)	0.38 (0.35; 0.56)	0.62 (0.39; 0.83)	*p* < 0.05
Jejunum (muscularis-serosa)	0.04 (0.01; 0.14)	0.12 (0.04;0.14)	n.s.
Ileum (mucosa-submucosa)	0.27 (0.2; 0.35)	0.49 (0.27; 0.9)	n.s.
Ileum (muscularis-serosa)	0.05 (0.02; 0.13)	0.07 (0.03; 0.23)	n.s.
Colon (mucosa-submucosa)	0.38 (0.27; 0.49)	0.45 (0.36; 0.86)	n.s.
Colon (muscularis-serosa)	0.02 (0.01; 0.07)	0.06 (0.04; 0.11)	n.s.
Gall bladder	0.34 (0.18; 0.44)	0.9 (0.74; 2.04)	*p* < 0.01
Urinary bladder	0.11 (0.08;0.41)	0.2 (0.16;0.6)	*p* < 0.01

*P*_*AW*_ airway pressure; Values are presented as median (lower quartile; upper quartile); tested on a randomized basis, Wilcoxon matched pairs test

For individual animal data see an additional file as an online supplement (Additional file 1).

## Discussion

We examined the influence of lung protective mechanical ventilation in an experimental ARDS model on systemic and regional blood flow using the colored microspheres method. Mechanical ventilation with limited inspiratory P_AW_ and moderate respiratory acidosis resulted in an increase in CO, and variable changes in tissue perfusion. The marked increase in adrenal perfusion indicates a sympatho-adrenal response to the moderate respiratory acidosis.

Our results confirm recent data on the interaction of lung protective mechanical ventilation and moderate respiratory acidosis on systemic hemodynamics [10] and extend these findings to the regional capillary and nutritional circulation. In our experimental animals limiting airway and intrathoracic pressures did not result in an increased preload as intrathoracic blood volume, central venous and pulmonary capillary wedge pressure remained unchanged. Along with the unchanged preload stroke volume only slightly increased. However, the combination of the slight increases in stroke volume and heart rate caused a significant increase in CO, mean arterial and pulmonary arterial pressure. Although we did not determine sympatho-adrenergic and thyroid hormones the increase both in adrenal and thyroid blood flows during ventilation with limited inspiratory P_AW_ may indicate that the augmented systemic and regional blood flow, at least in part, may have been caused by a hormonal response to moderate respiratory acidosis. In line with this observation hypercapnic acidosis has been previously reported to be attributed to sympathetic activation and catecholamine release [16–18] which, at least in states of mild to moderate hypercapnic acidosis, overrides the direct depressant effects of carbon dioxide on myocardial cells [18, 19] and precapillary resistance vessels [20].

In parallel with the increase in systemic blood flow regional myocardial perfusion of the right and left ventricle was markedly increased. This is in agreement with previous in vitro and in vivo data obtained during hypercapnia in coronary vessels [17, 21] and reflects the elevated metabolic demands of the myocardial pump associated with the increase in heart rate and CO. In our animals without coronary artery disease perfusion of both subepicardial and subendocardial perfusion rose to a similar extent and the transmural distribution of myocardial blood flow was not altered by the ventilatory strategy. In contrast, previous data obtained in animals with single-vessel coronary artery disease during severe hypercapnia of more than 120 mmHg showed redistribution of blood flow from the subendocardial to subepicardial myocardium [21]. Therefore, clinicians should be aware of potential negative effects of hypercapnia in patients with multivessel coronary artery disease and/or myocardial insufficiency who are prone to myocardial ischemia.

Although maintaining normocapnia is essential in patients with intracranial hypertension even patients with intracranial pathologies may benefit from lung protective mechanical ventilation [22]. In our study ventilation with limited inspiratory P_AW_ and moderate respiratory acidosis in the absence of intracranial hypertension resulted in a marked increase in cerebral and spinal perfusion. As we evaluated nutritional blood flow with the microsphere method our results may be of clinical relevance especially for patients at risk for cerebral and spinal ischemic lesions, patients with cerebral vasospasm due to subarachnoid hemorrhage and critically ill patients being prone for septic encephalopathy which has been claimed to be associated with an impairment of cerebral nutritional perfusion and metabolism [23, 24].

Mechanical ventilation may also compromise renal hemodynamics [25, 26]. In our experimental animals, despite an increase in systemic blood flow, renal perfusion did not improve during ventilation with limited inspiratory P_AW_ and moderate respiratory acidosis. This finding is consistent with observations of impaired renal blood flow due to renal vasoconstriction and an increase in renal vascular resistance in patients with hypercapnic respiratory failure [27, 28] and in patients with ARDS [29]. Therefore, during lung protective ventilation close monitoring of renal function is essential.

Mechanical ventilation has been suggested to contribute to the development of multiple organ dysfunction by potentiating adverse effects of underlying critical illness on splanchnic perfusion [30]. Because the mucosal-submucosal and muscularis-serosal layers of the intestinal wall have different metabolic and oxygen requirements [31], with the mucosa–submucosa representing the layer with the highest demand, we measured blood flow to the different layers separately. Irrespective of the applied ventilatory strategy, mucosal–submucosal exceeded muscularis–serosal perfusion in our experimental animals, which is in accordance with previous investigations in different mammalian species [31] and reflects the high metabolic activity of the intestinal mucosa. While we found no significant change in the regional perfusion of the stomach, ileum and colon, the blood flow to the esophageal and jejunal mucosa-submucosa, the esophageal and duodenal muscularis-serosa, the gallbladder, and urinary bladder was increased during ventilation with limited inspiratory P_AW_. Pancreatic perfusion remained unchanged and arterial hepatic perfusion even tended to decrease when inspiratory P_AW_ was limited. This observation may, at least in part, have been caused by the intrinsic hepatic arterial buffer response [32] which counter-regulates changes in portal-venous and hepatic arterial blood flow to maintain overall hepatic blood flow constant.

During ventilation with limited inspiratory P_AW_ the splenic perfusion also remained constant despite the increase in systemic blood flow. Although we were not able to determine the splenic weight during each ventilatory strategy the concomitant marked rise of the hemoglobin level suggests a splenic response to respiratory acidosis which has been shown to include a constriction of splenic vessels and depletion of erythrocytes from the splenic blood reservoir both in experimental animals [33] and humans [34].

### Methodology

We used the colored microsphere method which is an established method for blood flow measurements [12, 35]. The theoretical basis of the microsphere technique is analogous to that of the indicator-dilution method and the number of particles impacted in a given tissue is proportional to the volume of particle-containing blood perfusing that tissue. A major advantage of this method is its ability to quantitate nutritive blood flow to individual tissue regions which may be as small as separate layers of the intestinal wall [12] and that measurements are possible without surgical interventions such as implantation of electromagnetic flow probes which per se may alter regional perfusion. The validity of using microspheres to study regional blood flow distribution is based on adequate mixing of injected microspheres and even distribution to the various parts of the body. This was demonstrated in our study by the close correlation between the numbers of microspheres trapped in the reference blood samples from different sites in the aorta and nearly identical blood flows to both adrenal glands.

### Study limitations

Because direct measurement of regional perfusion is not possible in critical ill patients we used a porcine model of ARDS which was induced with oleic acid. Therefore, our findings are not generalizable to humans and may vary based on different etiologies of ARDS. Limiting inspiratory P_AW_ resulted in a decrease in minute ventilation and moderate permissive respiratory acidosis which is common in daily clinical practice. Consequently, due to the combined variation of intrathoracic pressures and acid base balance we were not able to separate the effects of both parameters on the systemic and regional blood flows. Although gas exchange variables stabilized during equilibration periods before each measurement the time limits of our study probably were too short to elucidate potential carry over effects due to the cross-over design, e.g. long-term effects on a transcriptional/translational level and slow acting counter-regulatory effects. At last, as we did not determine any vasoactive hormones, we could only speculate on additional hormonal effects on systemic and regional blood flows by the observation of marked increases in adrenal and thyroid perfusion.

## Conclusions

In this porcine model of ARDS mechanical ventilation with limited inspiratory P_AW_ associated with moderate respiratory acidosis resulted in an increase in systemic blood flow which was not uniformly directed to the regional vascular beds. Our results may add some physiological understanding to the complex field of heart-lung interactions and its´ effects on regional perfusion and may promote further clinical investigation with special attention to subgroups of patients, e.g. with cardiac, renal and cerebral pathologies.

## Additional file

Additional file 1: Individual animal data. Excel file with individual data of all animals (ventilation, gas exchange, cardiovascular, and regional blood flow variables). (XLSX 49 kb)

## Abbreviations

ARDS: Acute respiratory distress syndrome
CO: Cardiac output
FiO_2_: Inspiratory oxygen fraction
P_AW_: Airway pressure
V_T_: Tidal volume
